# Phylogenetic placement of the enigmatic parasite, *Polypodium hydriforme*, within the Phylum Cnidaria

**DOI:** 10.1186/1471-2148-8-139

**Published:** 2008-05-09

**Authors:** Nathaniel M Evans, Alberto Lindner, Ekaterina V Raikova, Allen G Collins, Paulyn Cartwright

**Affiliations:** 1 Department of Ecology and Evolutionary Biology, University of Kansas, Lawrence, Kansas 66045, USA; 2 CEBIMar, University of São Paulo, São Sebastião, Brazil; 3 Institute of Cytology of the Russian Academy of Sciences, St. Petersburg, Russia; 4 National Systematics Laboratory of NOAA Fisheries Service, National Museum of Natural History, Smithsonian Institution, Washington, DC 20013-7012, USA

## Abstract

**Background:**

*Polypodium hydriforme *is a parasite with an unusual life cycle and peculiar morphology, both of which have made its systematic position uncertain. *Polypodium *has traditionally been considered a cnidarian because it possesses nematocysts, the stinging structures characteristic of this phylum. However, recent molecular phylogenetic studies using 18S rDNA sequence data have challenged this interpretation, and have shown that *Polypodium *is a close relative to myxozoans and together they share a closer affinity to bilaterians than cnidarians. Due to the variable rates of 18S rDNA sequences, these results have been suggested to be an artifact of long-branch attraction (LBA). A recent study, using multiple protein coding markers, shows that the myxozoan *Buddenbrockia*, is nested within cnidarians. *Polypodium *was not included in this study. To further investigate the phylogenetic placement of *Polypodium*, we have performed phylogenetic analyses of metazoans with 18S and partial 28S rDNA sequences in a large dataset that includes *Polypodium *and a comprehensive sampling of cnidarian taxa.

**Results:**

Analyses of a combined dataset of 18S and partial 28S sequences, and partial 28S alone, support the placement of *Polypodium *within Cnidaria. Removal of the long-branched myxozoans from the 18S dataset also results in *Polypodium *being nested within Cnidaria. These results suggest that previous reports showing that *Polypodium *and Myxozoa form a sister group to Bilateria were an artifact of long-branch attraction.

**Conclusion:**

By including 28S rDNA sequences and a comprehensive sampling of cnidarian taxa, we demonstrate that previously conflicting hypotheses concerning the phylogenetic placement of *Polypodium *can be reconciled. Specifically, the data presented provide evidence that *Polypodium *is indeed a cnidarian and is either the sister taxon to Hydrozoa, or part of the hydrozoan clade, Leptothecata. The former hypothesis is consistent with the traditional view that *Polypodium *should be placed in its own cnidarian class, Polypodiozoa.

## Background

**Figure 1:**
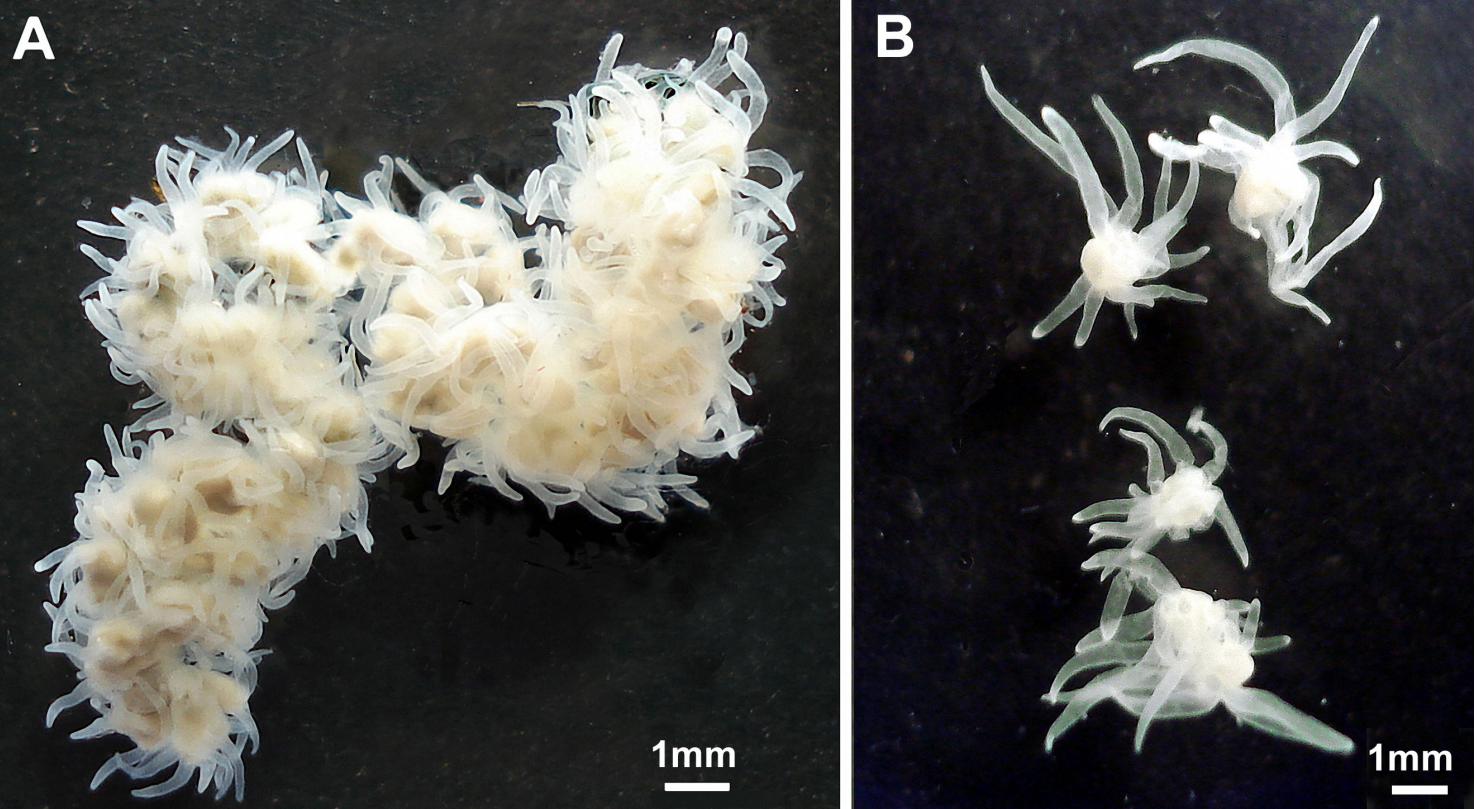
***Polypodium hydriforme*.** A) Stolon stage just after emerging from the host oocyte. B) Four specimens of free-living *Polypodium *with 12 tentacles. Photos by E. Raikova.

*Polypodium hydriforme *is an endocellular parasite whose unusual life cycle, peculiar morphology, and high rates of DNA evolution, have led to much controversy regarding its phylogenetic position within metazoans [1-5]. *Polypodium *spends most of its life inside the oocytes of acipenseriform fishes (sturgeons and paddlefish). During this time, *Polypodium *develops from a binucleate cell into an inside-out planuliform larva and then into an elongate inside-out stolon; the epidermal cell layer is located internal to the body and the gastrodermis is located externally [6-8]. The embryo, larva and stolon are surrounded by a protective polyploid cell, which also functions in digestion [7]. Just prior to host spawning, *Polypodium *everts to the normal position of cell layers, revealing tentacles scattered along the stolon. During eversion, the yolk of the host oocyte fills the gastral cavities of the parasite, supplying the future free-living stage with nutrients [6,7]. Finally, upon emerging from the host egg in fresh water, the free-living stolon (Figure 1A) fragments into individual medusoid-like forms (Figure 1B) that go on to multiply by means of longitudinal fission, form sexual organs, and ultimately infect host fish with their gametophores [6-9].

Two conflicting hypotheses have been proposed regarding the phylogenetic placement of *Polypodium*. The first, more traditional hypothesis is that *Polypodium *is a cnidarian. Some have suggested it is nested within a derived group of hydrozoans, the Narcomedusae [10-13] or the cnidarian class Scyphozoa [14]; while others have suggested it belongs to a separate cnidarian class, Polypodiozoa [1,15,16]. The assignment of *Polypodium *to Cnidaria is based primarily on morphological evidence, most notably the fact that *Polypodium *possesses nematocysts [17,18], the stinging structures characteristic of all cnidarians. In addition, the presence of tentacles and overall body-plan organization of *Polypodium *are reminiscent of cnidarians, although it is unclear if the adult free-living stage is homologous to a polyp or medusa stage. This hypothesis is supported by a cladistic analysis of small subunit nuclear ribosomal DNA (18S rDNA) sequences in conjunction with morphological characters (including nematocysts) [2]. In this study, *Polypodium *falls within the medusozoan clade of cnidarians, although the non-cnidarian placozoan, *Trichoplax *[19,20], also fell within this clade, rendering Cnidaria paraphyletic.

The second hypothesis is that *Polypodium *is the sister taxon to Myxozoa, a diverse group of parasites in aquatic animals, and that *Polypodium *+ Myxozoa is the sister group to Bilateria [2-4]. This hypothesis is derived from cladistic analyses utilizing 18S rDNA sequences [2-4]. However, because *Polypodium *and myxozoans have unusually high divergence rates in their 18S rDNA sequences, these cladistic analyses have been criticized by a number of authors who suggest that the data might be unduly affected by long-branch attraction (LBA) [5,21,22]. Despite some attempts to overcome the effects of LBA through the use of a maximum likelihood (ML) approach [21-23] and pruning long branches [5,22], these results have been largely silent on the placement of *Polypodium*. For instance, Kim et al. [22] applied a maximum likelihood approach to 18S rDNA sequence data and found that myxozoans and *Polypodium *did not group together. Instead, *Polypodium *was part of an unresolved polytomy that included several cnidarian lineages and *Trichoplax*, as well as myxozoans + Bilateria. Most recently, Jimenez-Guri et al. [24] utilized multiple protein-coding gene sequences in a ML analysis and found the myxozoan, *Buddenbrockia plumatellae *nested within cnidarians. Unfortunately, this study had relatively limited sampling of cnidarians and did not include *Polypodium*.

In an attempt to resolve this controversy, we sequenced an additional marker in *Polypodium*, a partial gene sequence of the large nuclear ribosomal unit (28S rDNA), and greatly expanded the taxonomic sampling of cnidarian sequences. Using this approach, we provide evidence that *Polypodium *is nested within Cnidaria and does not group with myxozoans.

## Results

### Sampled taxa

All taxa used in this study are arranged taxonomically in Table 1. 155 sequences were obtained from GenBank. 45 new cnidarian sequences for 18S and 59 for 28S (including 2 new 18S and 2 new partial 28S from *Polypodium *taxa) were generated for this study and deposited in GenBank (see Table 1 for accession numbers). *Polypodium hydriforme *sequences were obtained from both North American and Eurasian hosts. Eurasian samples were collected from two individuals of *Acipenser ruthenus*. North American samples were collected from *Polyodon spathula *and *Scaphirhynchus platorynchus*. This is the first reported presence of *Polypodium *infection in *Scaphirhynchinae*. While *Polypodium *was recovered from the oocytes of *S. platorynchus*, the sample from which we extracted sequence data was found externally attached to its presumed host. More specific collection data for *Polypodium *specimens are associated with each sequence submitted to GenBank (see Table 1 for accession numbers).

**Table 1:** Taxon and sequence list

		Accession numbers		
			
Higher classification	Taxon ID	28S	18S	Voucher
**Bilateria**				
Annelida	*Proceraea cornuta*	AF212165	AF212179	
Annelida	*Urechis caupo*	AF342804	AF342805	
Arthropoda	*Limulus polyphemus*	AF212167	U91490	
Arthropoda	*Tenebrio *sp.*/Tenebrio molitor*	AY210843	X07801	
Brachiopoda	*Phoronis vancouverensis*	AF342797	U12648	
Chordata	*Oncorhynchus *sp.*/O. kisutch*	U34341	AF030250	
Chordata	*Petromyzon marinus*	AF061798	M97575.1	
Chordata	*Raja schmidti*	AF278683	AF278682	
Chordata	*Triakis semifasciata*	AF212182	AF212180	
Echinodermata	*Strongylocentrotus purpuratus*	AF212171	L28056.	
Hemichordata	*Cephalodiscus gracilis*	AF212172	AF236798	
Hemichordata	*Harrimania *sp.	AF212173	AF236799	
Hemichordata	*Ptychodera flava*	AF212176	AF278681	
Hemichordata	*Ptychoderidae*	AF278684	D14359	
Hemichordata	*Saccoglossus kowalevskii*	AF212175	L28054	
Kinorhyncha	*Pycnophyes *sp.*Tjarno*	AY859597	AY859598	
Mollusca	*Parvicardium minimum*	DQ279966	DQ279942	
Mollusca	*Placopecten magellanicus*	AF342798	X53899	
Nematoda	*Caenorhabditis elegans*	X03680	X03680	
Nematomorpha	*Chordodes morgani*	AF342787	AF036639	
Nemertea	*Amphiporus *sp.	AF342786	AF119077	
Nemertodermatida	*Meara stichopi*	AY157605	AF119085	
Onychophora	*Peripatus *sp.	AY210836	AY210837	
Platyhelminthes	*Diclidophora denticulata*	AY157169	AJ228779	
Platyhelminthes	*Stenostomum leucops*	AY157151	D85095	
Platyhelminthes	*Stylochus zebra*	AF342800	AF342801	
Priapulida	*Priapulus caudatus*	AY210840	Z38009	
Sipuncula	*Phascolopsis gouldii*	AF342795	AF342796	
Tardigrada	*Milnesium*.sp.\*M. tardigradum*	AY210826	U49909	
Urochordata	*Styela plicata*	AF158724	L12444	
Urochordata	*Thalia democratica*	AF158725	D14366	
**Cnidaria**				
Polypodiozoa	*Polypodium (Host: Acipenser ruthenus)*	EU272585	EU272630	
Polypodiozoa	*Polypodium (Host: Polyodon spathula)*		EU272629	
Polypodiozoa	*Polypodium (Host:Scaphirhynchus platorynchus)*	EU272586		
Anthozoa, Antipatharia	*Antipathes galapagensis*	AY026365	AF100943	
Anthozoa, Scleractinia	*Montastraea franksi*	AY026375	AY026382	
Cubozoa, Carybdeidae	*Carybdea rastonii*	AY920787	AF358108	
Cubozoa, Carybdeidae	*Darwin carybdeid *sp.	AY920788	AF358105	
Cubozoa, Carybdeidae	*Tripedalia cystophora*	EU272595	EU272637	
Cubozoa, Chirodropidae	*Chironex fleckeri*	AY920785	AF358104	
Cubozoa, Chirodropidae	*Chiropsalmus *sp.	AY920786	AF358103	
Hydrozoa, Capitata	*Dipurena ophiogaster*	EU272560	EU272615	KUNHM 2803
Hydrozoa, Capitata	*Ectopleura dumortieri*	EU272561	EU272616	
Hydrozoa, Capitata	*Euphysora bigelowi*	EU272563	EU272618	KUNHM 2829
Hydrozoa, Capitata	*Moerisia *sp.	AY920801	AF358083	
Hydrozoa, Capitata	*Pennaria disticha*	EU272581	AY920762	
Hydrozoa, Capitata	*Polyorchis penicillatus*		AF358090	
Hydrozoa, Capitata	*Porpita *sp.	AY920803	AF358086	
Hydrozoa, Capitata	*Ralpharia gorgoniae*	EU272590	EU272633	KUNHM 2778
Hydrozoa, Capitata	*Scrippsia pacifica*	AY920804	AF358091	
Hydrozoa, Capitata	*Solanderia ericopsis*	EU272593	EU272636	MHNG INVE29593
Hydrozoa, Capitata	*Velella *sp.	EU272597	AF358087	
Hydrozoa, Capitata	*Zanclea prolifera*	EU272598	EU272639	KUNHM 2793
Hydrozoa, Capitata	*Zyzzyzus warreni*	EU272599	EU272640	KUNHM 2777
Hydrozoa, Capitata	*Candelabrum cocksii*	AY920796	AY920758	MHNG INVE29531
Hydrozoa, Capitata	*Cladocoryne floccosa*	EU272551	EU272608	
Hydrozoa, Filifera	*Bimeria vestita*	EU272548	EU272605	
Hydrozoa, Filifera	*Bougainvillia carolinensis*	EU272549	EU272606	
Hydrozoa, Filifera	*Brinckmannia hexactinellidophila*	EU272550	EU272607	MHNG INVE38148
Hydrozoa, Filifera	*Clava multicornis*	EU272552	EU272609	
Hydrozoa, Filifera	*Clavactinia gallensis*	EU272553	EU272610	MHNG INVE33470
Hydrozoa, Filifera	*Cordylophora caspia*	EU272556	EU272612	
Hydrozoa, Filifera	*Corydendrium *sp.	EU272557	EU272613	KUNHM 2764
Hydrozoa, Filifera	*Dicoryne conybearei*	EU272559	EU272614	MHNG INVE32949
Hydrozoa, Filifera	*Eudendrium.racemosum*	EU272562	EU272617	
Hydrozoa, Filifera	*Fabienna sphaerica*	AY920797	AY920767	
Hydrozoa, Filifera	*Garveia annulata/Garveia *sp.	EU272564	AY920766	KUNHM 2860
Hydrozoa, Filifera	*Hydra circumcincta*	AY026371	AF358080	
Hydrozoa, Filifera	*Hydractinia symbiolongicarpus*	EU272568	EU272621	
Hydrozoa, Filifera	*Hydrichthella epigorgia*	EU272569	EU272622	KUNHM 2665
Hydrozoa, Filifera	*Hydrichthys boycei*	EU272570		MHNG INVE37417
Hydrozoa, Filifera	*Koellikerina fasciculata*	EU272571	EU272623	
Hydrozoa, Filifera	*Leuckartiara octona*	EU272573	EU272624	
Hydrozoa, Filifera	*Lizzia blondina*	EU272574	EU272625	
Hydrozoa, Filifera	*Pachycordyle pusilla*	EU272579	EU272627	MHNG INVE32953
Hydrozoa, Filifera	*Pandea *sp.	EU272580	AY920765	
Hydrozoa, Filifera	*Podocoryne carnea*	AY920802	AF358092	
Hydrozoa, Filifera	*Proboscidactyla ornata*	EU272587	EU272631	KUNHM 2767
Hydrozoa, Filifera	*Pruvotella grisea*	EU272588	EU272632	MHNG INVE34436
Hydrozoa, Filifera	*Rathkea octopunctata*	EU272591	EU272634	KUMIP 314321
Hydrozoa, Filifera	*Rhizogeton nudus*	EU272592	EU272635	MHNG INVE35757
Hydrozoa, Filifera	*Turritopsis dohrnii*	EU272596	EU272638	MHNG INVE29753
Hydrozoa, Leptothecata	*Abietinaria filicula*	EU272540	EU272600	MHNG INVE29947
Hydrozoa, Leptothecata	*Aglaophenia tubiformis*	EU272543	EU272601	MHNG INVE29967
Hydrozoa, Leptothecata	*Amphisbetia minima*	EU272544	EU272602	MHNG INVE25071
Hydrozoa, Leptothecata	*Anthohebella parasitica*	EU272545	EU272603	MHNG INVE29762
Hydrozoa, Leptothecata	*Clytia noliformis*	EU272554	EU272611	
Hydrozoa, Leptothecata	*Halecium muricatum*	EU272565	EU272619	MHNG INVE29028
Hydrozoa, Leptothecata	*Halopteris minuta*	EU272567	EU272620	MHNG INVE25073
Hydrozoa, Leptothecata	*Melicertum octocostatum*	EU272575	AY920757	USNM 1073342
Hydrozoa, Leptothecata	*Octophialucium indicum*	EU272577	EU272626	MHNG INVE29970
Hydrozoa, Leptothecata	*Plumularia setacea*	EU272583	EU272628	MHNG INVE36298
Hydrozoa, Siphonophorae	*Agalma elegans*	EU272542	AY937313	YPM 35029
Hydrozoa, Siphonophorae	*Apolemia *sp.	EU272546	AY937331	YPM 35090
Hydrozoa, Siphonophorae	*Cordagalma cordiforme*	EU272555	AY937317	YPM 35032
Hydrozoa, Siphonophorae	*Halistemma rubrum*	EU272566	AY937358	YPM 35359
Hydrozoa, Siphonophorae	*Nanomia bijuga*	EU272576	AY937338	YPM 35043
Hydrozoa, Siphonophorae	*Nectopyramis *sp.	AY026377	AF358068	
Hydrozoa, Siphonophorae	*Physophora hydrostatica*	EU272582	AY937342	YPM 35046
Hydrozoa, Siphonophorae	*Sulculeolaria quadrivalvis*	EU272594	AY937353	YPM 35357
Hydrozoa, Stylasteridae	*Crypthelia cryptotrema*	EU272558	EU272641	USNM1027758
Hydrozoa, Stylasteridae	*Lepidopora microstylus*	EU272572	EU272644	USNM1027724
Hydrozoa, Stylasteridae	*Pseudocrypthelia pachypoma*	EU272589	EU272643	USNM1027728
Hydrozoa, Stylasteridae	*Adelopora crassilabrum*	EU272541	EU272642	USNM1027760
Hydrozoa, Trachylina	*Limnocnida tanganyicae*	AY920795	AY920755	
Hydrozoa, Trachylina	*Maeotias marginata*	EU247810		
Hydrozoa, Trachylina	*Olindias phosphorica*	EU247808	AY920753	MHNG INVE29811
Scyphozoa, Coronatae	*Atolla vanhoeffeni*	AY026368	AF100942	
Scyphozoa, Coronatae	*Nausithoe rubra*	AY920776	AF358095	
Scyphozoa, Rhizostomea	*Catostylus *sp.	AY920777	AF358100	
Scyphozoa, Semaeostomeae	*Chrysaora melanaster*	AY920780	AF358099	
Scyphozoa, Semaeostomeae	*Aurelia *sp.	EU272547	EU272604	
Scyphozoa, Semaeostomeae	*Phacellophora camtschatica*	AY920778	AF358096	
Staurozoa, Stauromedusae	*Craterolophus convolvulus*	AY920781	AY845344	
Staurozoa, Stauromedusae	*Haliclystus octoradiatus*	AH014894	AY845346	
Staurozoa, Stauromedusae	*Haliclystus sanjuanensis*	AY920782	AF358102	
**Myxozoa**				
Malacosporea	*Buddenbrockia plumatellae*		AJ937883	
Myxosporea	*Henneguya salminicola*	AY302726		
Myxosporea	*Kudoa trifolia*	AM490336	AM183300	
Myxosporea	*Kudoa unicapsula*	AM490335	AM490334	
Myxosporea	*Myxobolus cerebralis*		EF370481	
Myxosporea	*Myxobolus dogieli*		EU003978	
Myxosporea	*Parvicapsula limandae*		EF429096	
**Outgroups**				
Choanoflagellida				
Codonosigidae	*Monosiga brevicollis*	AY026374	AF084618	
Salpingoecidae	*Salpingoeca infusionum*	AY026380	AF100941	
Ctenophora,				
Cyclocoela	*Beroe ovata*	AY026369	AF293694	
Cyclocoela	*Mnemiopsis leidyi*	AY026373	AF293700	
Typhlocoela	*Pleurobrachia bachei*	AY026378	AF293677	
Fungi				
Ascomycota	*Candida albicans*	X70659	X53497	
Ascomycota	*Saccharomyces cerevisiae*	J01355	M27607	
Basidiomycota	*Tricholoma matsutake*	U62964	U62538.1	
Mucoromycotina	*Mucor racemosus*	AJ271061	AJ271061	
Porifera,				
Calcarea	*Leucosolenia *sp.	AY026372	AF100945	
Demospongia	*Mycale fibrexilis*	AY026376	AF100946	
Demospongia	*Suberites ficus*	AY026381	AF100947	

A complete list of sequences used in the analyses with GenBank accession numbers and museum voucher numbers. Bold numbers indicate new sequences generated for this study. KUMIP = University of Kansas Museum of Invertebrate Paleontology, KUNHM = University of Kansas Natural History Museum, MHNG = Muséum d'histoire naturelle de Genève, YPM = Yale Peabody Museum, USNM = US National Museum of Natural History.

All *Polypodium *sequences were newly generated for this study. We did not include the previously published 18S *Polypodium *sequence (GenBank accession number U37526) because of concern over the quality of the sequence which included a number of ambiguities. Furthermore, while the two new *Polypodium *18S sequences (from hosts *Acipenser ruthensus *and *Polyodon spathula*) differed from each other by a total of 8 sites they differed from #U37526 by 77 and 83 sites respectively. These differences included a large number of insertions and deletions. The two new 28S sequences (from hosts *Acipenser ruthensus *and *Scaphirhynchus platorynchus*) only differed from each other by 2 sites.

### Position of *Polypodium*

**Figure 2:**
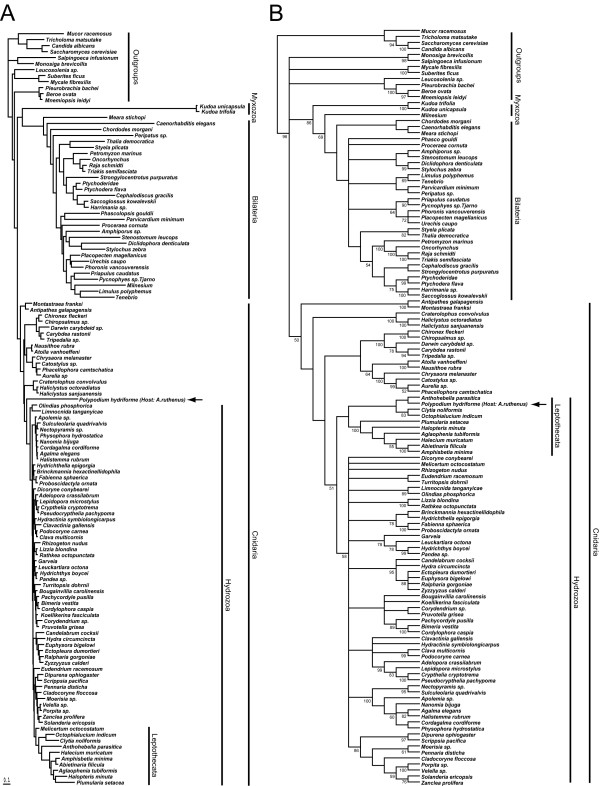
**Phylogenetic hypotheses of relationships among 126 metazoan taxa, based on a combined analysis of nearly complete 18S and partial 28S rDNA sequences.** Arrow indicates *Polypodium *taxa. A) Maximum likelihood topology. The assumed model (GTR+I + G) has six substitutions rates estimated from the data (A-C, 1.1786; A-G, 3.3654; A-T, 1.7283; C-G, 0.7403; C-T, 4.7803; G-T, 1.0000), an assumed proportion of invariant sites (0.1692) and a gamma shaped parameter or (0.5584). The length of the bar indicates 0.1 substitutions per site. Bootstrap values for this topology are indicated on the cladogram in Additional file 1. B) Strict consensus of 32 trees of length 25141 from a parsimony analyses. Bootstrap values of 50 or greater are indicated.

The complete combined dataset of 18S rDNA and partial 28S rDNA contains 4842 characters, 2901 of which are variable and 2124 parsimony informative. Both the ML and parsimony topologies reconstructed from the combined dataset suggest that *Polypodium *is nested within a monophyletic Cnidaria, and myxozoans are the sister taxon to bilaterians (Figure 2). The ML bootstrap values supporting a monophyletic Cnidaria (including *Polypodium*), a monophyletic Medusozoa (including *Polypodium*) and the *Polypodium *+ hydrozoan clade are 73, 67 and 73 respectively (Figure 2A, and Additional file 1). Parsimony analysis of the combined dataset differs from that of ML in that *Polypodium *is nested within a group of hydrozoans, the leptothecates (Figure 2B). The parsimony bootstrap values supporting a monophyletic Cnidaria and Hydrozoa, with *Polypodium *nested within these clades are 50 and 51 respectively (Figure 2B). The clade nested within hydrozoans, that includes *Polypodium *+ leptothecates is weakly supported in the sub-sampling tests with a bootstrap value of less than 50.

**Figure 3:**
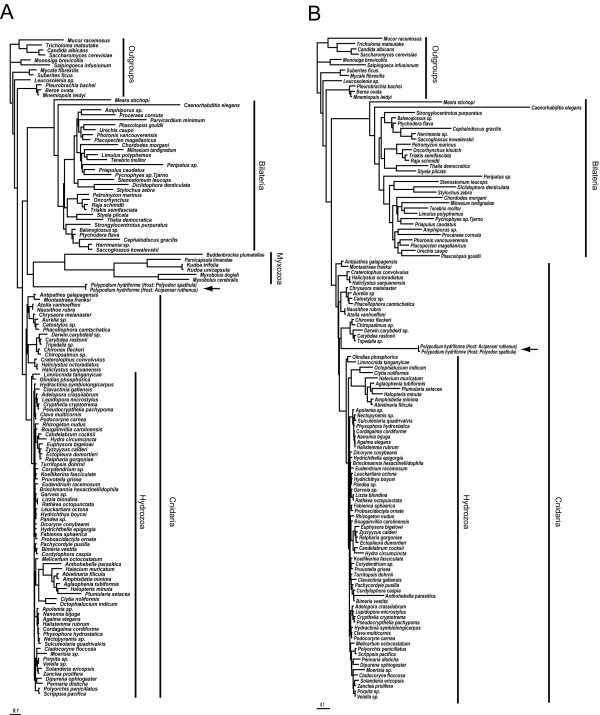
**ML topologies of metazoan relationships of nearly complete 18S rDNA sequences.** Arrow indicates *Polypodium *taxa. Bootstrap values for both topologies are indicated on the cladograms in Additional file 3. A) 132 taxa including 6 myxozoan taxa and two *Polypodium *taxa. The assumed model (GTR+I + G) has six substitutions rates estimated from the data (A-C, 1.4071; A-G, 3.3470; A-T, 1.6901; C-G, 0.84888; C-T, 4.7638; G-T, 1.0000), an assumed proportion of invariant sites (0.1757) and a gamma shaped parameter or (0.5837). B) Same dataset as (A) but with the 6 myxozoan taxa removed. The assumed model (GTR+I + G) has six substitutions rates estimated from the data (A-C, 1.4115; A-G, 3.3559; A-T, 1.7502; C-G, 0.8342; C-T, 4.8554; G-T, 1.0000), an assumed proportion of invariant sites (0.2464) and a gamma shaped parameter or (0.6326). The length of the bar indicates 0.1 substitutions per site.

The analyses using partial 28S rDNA sequences alone (129 sampled taxa) contains 1756 characters, 1196 of which are parsimony informative. The ML topology using this dataset reveals *Polypodium *nested within Cnidaria, specifically within leptothecate hydrozoans, (Additional file 2). This analysis however fails to recover a monophyletic Cnidaria, as the anthozoans are placed outside the Cnidaria + Bilateria clade. Analysis of the 18S rDNA dataset alone (132 taxa, 3038 characters, 1469 parsimony informative) under both optimality criteria conflicts with the combined and partial 28S topologies. The 18S rDNA topology for both criteria place *Polypodium *at the base of Bilateria (Figure 3A, Additional files 3, 4 and 5). However, the ML topology also reflects a sister relationship between *Polypodium *and myxozoans (Figure 3A and Additional file 3A) while the parsimony topology does not (Additional files 4 and 5). Moreover, under parsimony criteria the position of myxozoans is dependent upon how gaps are coded: if gaps are coded as a fifth character state, myxozoans are placed as a highly derived clade of bilaterians (Additional file 4); if gaps are coded as missing, myxozoans are placed as sister to all metazoans (Additional file 5). The 18S analysis showing placement of *Polypodium *with Bilateria, and more specifically as sister to myxozoans, is consistent with previously reported studies using the same marker [2-4], but raises similar concerns of long-branch attraction [5].

### Test of long-branch attraction

Myxozoans and *Polypodium *have unusually high rates of evolution in their 18S and 28S rDNA sequences relative to the other sampled taxa. To investigate the influence of myxozoans on the placement of *Polypodium*, we removed the myxozoans from our three datasets and re-ran each analysis. Under the ML analysis of 18S rDNA, the removal of myxozoans results in the placement of *Polypodium *nested within Cnidaria (Figure 3B and Additional file 3B). This result suggests that the placement of *Polypodium *at the base of bilaterians in the 18S analysis (Figure 3A) was indeed an artifact of LBA. The placement of *Polypodium *within Cnidaria was not effected by the removal of myxozoans in the 28S (Additional file 6) and combined datasets (Additional file 7).

To investigate the possible role of LBA on myxozoan placement, we removed *Polypodium *from the combined ML analyses and found that it did not affect the position of Myxozoa at the base of the Bilateria (not shown). Given that bilaterians also form long branches, we tried removing all bilaterian sequences in the combined ML analysis. This resulted in a Myxozoa + *Polypodium *clade nested within Cnidaria (not shown). However, when *Polypodium *and bilaterians were removed, myxozoans fell outside the cnidarians (not shown). Similar effects of myxozoan placement to long-branches were also found in parsimony analyses of the combined dataset (not shown).

## Discussion

### *Polypodium *is a cnidarian

Our metazoan dataset of 18S and partial 28S rDNA sequences, with a large taxonomic sample of cnidarians, places *Polypodium *within a monophyletic Cnidaria. This accords with the fact that *Polypodium *possesses nematocysts [17,18] and a cnidarian-like body plan [7-9,12]. The precise placement of *Polypodium *within Cnidaria is less certain. The ML combined analysis places *Polypodium *as sister to Hydrozoa (Figure 2A), a hypothesis consistent with the suggestion that *Polypodium *be considered a separate class of cnidarians, Polypodiozoa [1]. By contrast, the combined parsimony analysis (Figure 2B) and the ML analyses of 28S alone (Additional file 2 and 6) place *Polypodium *within the hydrozoan clade Leptothecata. Given that leptothecates have relatively high rates of evolution within hydrozoans, one possible explanation for the conflicting hypotheses is that the placement of *Polypodium *within leptothecates is an artifact of LBA and that the combined data, in conjunction with the ML approach (Figure 2A), overcame this localized LBA artifact.

### Evolution of *Polypodium *life-history characters

Although the fresh water habitat of *Polypodium *is unusual for cnidarians, it is not unheard of, especially within hydrozoans. For instance, the model organism *Hydra *and the jellyfish *Craspedacusta *are both exclusively fresh-water hydrozoans. *Hydra *and *Craspedacusta *are distantly related [25] and our analyses do not indicate a close phylogenetic affinity of *Polypodium *to either of the clades containing these taxa. Thus, it appears that in the evolution of cnidarians, invasion to fresh-water habitats has happened at least three separate times.

Although *Polypodium *is the only known intracellular cnidarian parasite, other cnidarians have adopted parasitic life-styles [11,26-29]. For example, parasites belonging to the Narcomedusae (Hydrozoa) have been reported to live in the stomach cavities of other narcomedusae [11,27] and anthomedusae [27]. In addition, the sea anemone *Edwardsiella lineata *parasitizes the stomach cavity of the ctenophore *Mnemiopsis leidyi *[28] and the anemone *Peachia quinquecapitata *is reported to parasitize the stomachs of hydromedusa [29].

### Effects of long-branch attraction

The well-documented effects of long-branch attraction artifacts (reviewed in Bergsten [30]) are particularly concerning when investigating relationships amongst early-diverging metazoans, where rates between lineages vary greatly [22]. Suggestions for avoiding LBA artifacts include choice of appropriate markers [31,32], increased taxonomic sampling to effectively break up long branches [33,34] and utilization of best-fit models that incorporate rate variation [21-23]. Previous conflicting reports that show *Polypodium *and myxozoans form a sister taxon to Bilateria [2-4] can be explained by limited taxon sampling and an inadequate number of informative characters in their analyses, both of which confound long-branch problems. In this study, the increased taxonomic sampling of cnidarians and the addition of 28S rDNA sequence data proved critical to placing the highly divergent *Polypodium *taxon within Cnidaria. The choice of optimality criteria (ML vs. parsimony) both supported *Polypodium *as a cnidarian but did affect the placement within Cnidaria.

### *Polypodium *and Myxozoa

Our analyses are inconclusive in the placement of Myxozoa within metazoans. We found that myxozoans consistently grouped with long-branched taxa and that removal of long-branches resulted in myxozoans being placed to the next longest branch. For example myxozoans group with *Polypodium *in the absence of Bilateria and group with Bilateria in the absence of *Polypodium *(not shown).

Jimenez-Guri et al. [24] sampled the myxozoan, *Buddenbrockia*, and found it to fall within Cnidaria, as the sister group to two hydrozoan representatives and a single scyphozoan. Previous studies have suggested a sister group relationship between cnidarians and myxozoans [2-4], and some morphological evidence has been used to support this view [35]. Although our present study does not support this relationship, further investigation is merited. Myxozoans are a highly diverse group (reviewed in Kent et al. [36]) that comprise two clades, the Myxosporea and the Malacosporea [37]. We were only able to include 28S rDNA sequences from myxosporeans, although the malacosporean *Buddenbrockia *was included in our 18S analysis and found to group with other myxozoans and outside of Cnidaria. Future studies with a comprehensive sampling of myxozoans together with *Polypodium*, in a dataset that includes a large taxonomic sampling of cnidarians, should shed further light on the relationships between myxozoans and *Polypodium*.

## Conclusion

Although previous molecular phylogenetic hypotheses conflicted with the traditional interpretation of cnidarian affinity for *Polypodium*, the molecular evidence we present, using an augmented dataset, ultimately confirms and reconciles this traditional hypothesis and suggests that *Polypodium *is indeed a cnidarian. This study also reaffirms the importance to large taxonomic sampling and inclusion of additional informative characters for avoiding long-branch attraction artifacts.

## Methods

### DNA isolation, amplification and sequencing

Genomic DNA was extracted using Qiagen DNeasy kits according to manufacturer's protocol (QIAGEN Inc., Mississauga, ON) or a standard phenol/chloroform protocol. The latter method involved tissue digestion with proteinase K (20 mg/ml) in a lysis buffer (20 mM Tris-CL pH 8.0, 5 mM EDTA pH 8.0, 400 mM NaCl, 2%SDS), extraction with phenol/chloroform (1:1), precipitation with 2.5 vol. 95% EtOH, and elution in TE or H_2_O.

An approximately 1.8 kb portion of the gene coding for 18S was amplified and sequenced with universal eukaryotic primers as described by Medlin et al. [38], with the annealing temperature modified to 57°C. With the exception of *Polypodium *samples, a nearly complete, roughly 3 kb portion of the gene coding for 28S was amplified and sequenced with an approach modified from that reported in Collins et al. [25]. 28S was directly amplified in two fragments with combinations of primers F63mod+R2077sq and F1379+R3264 from Medina et al. [39] or newly developed medusozoan specific primers F97+R2084 and F1383+R3238 (F97: CCYYAGTAACGGCGAGT, R2084: AGAGCCAATCCTTTTCC, F1383: GGACGGTGGCCATGGAAGT, and R3238: SWACAGATGGTAGCTTCG). Amplifications of 28S were conducted with the following thermal profile: 4 minutes at 94°C; 30 cycles of 30 seconds at 94°C, 1 minute at 45°C, and 3 minutes at 72°C; and 10 minutes at 72°C. For *Polypodium*, a portion of the 5' end of 28S (approx. 0.8–1.0 kbps) was amplified using two universal metazoan primers (fw1and rev2) as reported by Sonnenberg et al. [40]. Sequencing was carried out using amplification primers and F635sq and R635sq from Medina et al. [39].

All gene fragments were purified and sequenced by Cogenics, Inc. (Houston, TX) and assembled and edited using Sequencher v4.5 (Gene Code Co., 2005). Sequences for each marker were aligned using the program MUSCLE [41]. The 28S sequence alignment was then trimmed to reflect only that region which included sequence data for *Polypodium*. This trimmed 28S dataset was analyzed separately and used in conjunction with the complete 18S sequences to create the combined dataset.

### Phylogenetic analyses

Phylogenetic analyses were performed using both maximum likelihood (ML) and parsimony criteria. ML searches were performed using GARLI v0.951.OsX-GUI [42] under an assumed GTR model with rates estimated from the data. The assumed model of nucleotide substitution was selected by using the Akaike Information Criterion (AIC) as implemented in ModelTest [43]. Each run was repeated 10 times from random starting trees using default termination conditions. Each run gave identical topologies and similar likelihood scores. 100 bootstrap replications were run in GARLI v0.951.0sX-GUI [42] under the same parameters.

To assess the effect that omitting length-variable regions has on topology, we removed these regions from the combined dataset, using the less stringent settings of Gblocks [44]. This dataset contained 126 metazoan taxa, 2415 characters, 1391 of which are parsimony informative. We found that removal of length-variable regions had no effect on the placement of *Polypodium *and minimal effect on overall topology in our combined ML analyses (Additional file 8). Therefore we performed all other analyses with the complete datasets, including the more variable regions.

Parsimony analyses were performed using TNTv.1.1 [45]. Separate tree searches were performed with gaps coded as missing and gaps coded as a fifth state. However, with one exception (see results for myxozoan placement with 18S data) there was no significant difference in topology. Numerous search methods available in TNT were utilized to search the tree space but the following approach was found to consistently recover trees with minimum lengths from our datasets. The implemented search was a driven new technology search with a random seed of 0 (where 0 = time). Default settings for sectorial searches (RSS and CSS) and tree fusing were used [46], with 5 replicates per repetition, and a requirement that the global optimum be found 20 times. TBR branch swapping was performed on the resulting trees and a strict consensus was calculated. TNT was used to calculate standard bootstrap values (1000 replicates). Alignments and trees for 18S, 28S and combined datasets have been submitted to TreeBASE http://www.treebase.org/treebase/index.html.

## Authors' contributions

This study was inspired by the work of EVR and originally conceived by EVR, AL and AGC. NME performed most of the data collection and submission of the new sequences. NME and PC performed the analyses. PC took the lead in organizing the study and drafting the manuscript with contributions from NME. All other authors provided comments and suggestions and approved the final manuscript.

## Supplementary Material

Additional file 1 ML topology of relationships based on combined data. ML topology identical to Figure 1A but as a cladogram showing bootstrap values. Click here for file

Additional file 2 ML topology of relationships based on partial 28S rDNA sequences. Relationships of 128 metazoan taxa were analyzed with partial 28S rDNA sequences. Click here for file

Additional file 3 ML topologies of relationships based on 18S data with and without myxozoans. ML topologies identical to Figure 3 but as cladograms showing bootstrap values. Click here for file

Additional file 4 Parsimony topology of relationships based on 18S rDNA sequences. This parsimony analysis of 18S rDNA sequences included 132 taxa with gaps coded as a fifth state. Click here for file

Additional file 5 Parsimony topology of relationships based on 18S rDNA sequences. This parsimony analysis of 18S rDNA sequences included 132 taxa with gaps coded as missing. Click here for file

Additional file 6 ML topology of relationships excluding myxozoans, based on partial 28S rDNA data. This ML analysis of partial 28S rDNA sequences excluded myxozoan taxa. Click here for file

Additional file 7 ML topology of relationships excluding myxozoans, based on combined data. This ML analysis of partial 28S rDNA and 18S sequences excluded myxozoan taxa. Click here for file

Additional file 8 ML topology of relationships based on combined data. This analysis of 126 metazoan taxa was based on combined 18S and partial 28S rDNA with length variable sequences removed. Click here for file
