# Correction: Phylogenetic placement of the enigmatic parasite, *Polypodium hydriforme*, within the Phylum Cnidaria

**DOI:** 10.1186/1471-2148-9-165

**Published:** 2009-07-15

**Authors:** Nathaniel M Evans, Alberto Lindner, Ekaterina V Raikova, Allen G Collins, Paulyn Cartwright

**Affiliations:** 1 Department of Ecology and Evolutionary Biology, University of Kansas, Lawrence, Kansas 66045, USA; 2 CEBIMar, University of São Paulo, São Sebastião, Brazil; 3 Institute of Cytology of the Russian Academy of Sciences, St. Petersburg, Russia; 4 National Systematics Laboratory of NOAA Fisheries Service, National Museum of Natural History, Smithsonian Institution, Washington, DC 20013-7012, USA

## Abstract

Correction to Evans, N.M., Lindner, A., Raikova, E.V., Collins, A.G. and Cartwright, P. Phylogenetic placement of the enigmatic parasite, *Polypodium hydriforme*, within the phylum Cnidaria. BMC Evol Biol, 2008, 8:139.

## Correction

Following the publication of this study [1] it was brought to our attention that the partial 28S rDNA sequence for *Polypodium hydriforme *used in some of our reported analyses was in fact a contaminant of an undetermined cnidarian species belonging to the genus *Obelia*. This contaminant was an isolated case involving a small batch of reagents and we are confident that no other sequences published in this work [1] are suspect. The 18S rDNA sequences were obtained in two laboratories using different reagents and different genomic DNA preparations derived from distinct tissue samples and are similar to a previously published 18s sequence from *Polypodium*.

The results of our 28S rDNA analyses (Additional file Two and Six, [1]) and our combined 18S and 28S rDNA analyses (Figure Two, Additional files One and Seven [1]) should be disregarded. We have reanalyzed the combined dataset including the *Polypodium *18S and *Obelia *28S as separate samples. The topologies with regards to the placement of *Polypodium *are consistent with those of our 18S rDNA only analyses (Figure ThreeB and Additional file ThreeB [1]).

Although the 18S rDNA analyses alone, excluding the long branch attractor Myxozoa, support our major conclusion that *Polypodium hydriforme *is a cnidarian (Figure ThreeB and Additional file ThreeB [1]) this placement should be viewed as tentative due to low bootstrap support (83% supporting a monophyletic Cnidaria that includes *Polypodium*) (Additional file ThreeB [1]). Further investigation of both the placement of *Polypodium *and the placement of Myxozoa within Metazoa are warranted.

We regret that this error was not discovered before publication and we apologize for any inconvenience this has caused. We gratefully acknowledge Maximiliano Maronna and Dr. Antonio Marques who, being the first to sequence 28S rDNA from *Obelia*, graciously brought this matter to our attention.
