# Supplementary material for: Phylogenetic placement of the enigmatic parasite, Polypodium hydriforme, within the Phylum Cnidaria
Source: BMC Evol Biol. 2008 May 9;8:139. doi: 10.1186/1471-2148-8-139 (PMC2396633; doi:10.1186/1471-2148-8-139)
Supplement: Additional file 1 — ML topology of relationships based on combined data. ML topology identical to Figure 1A but as a cladogram showing bootstrap values. [file 1471-2148-8-139-S1.pdf]

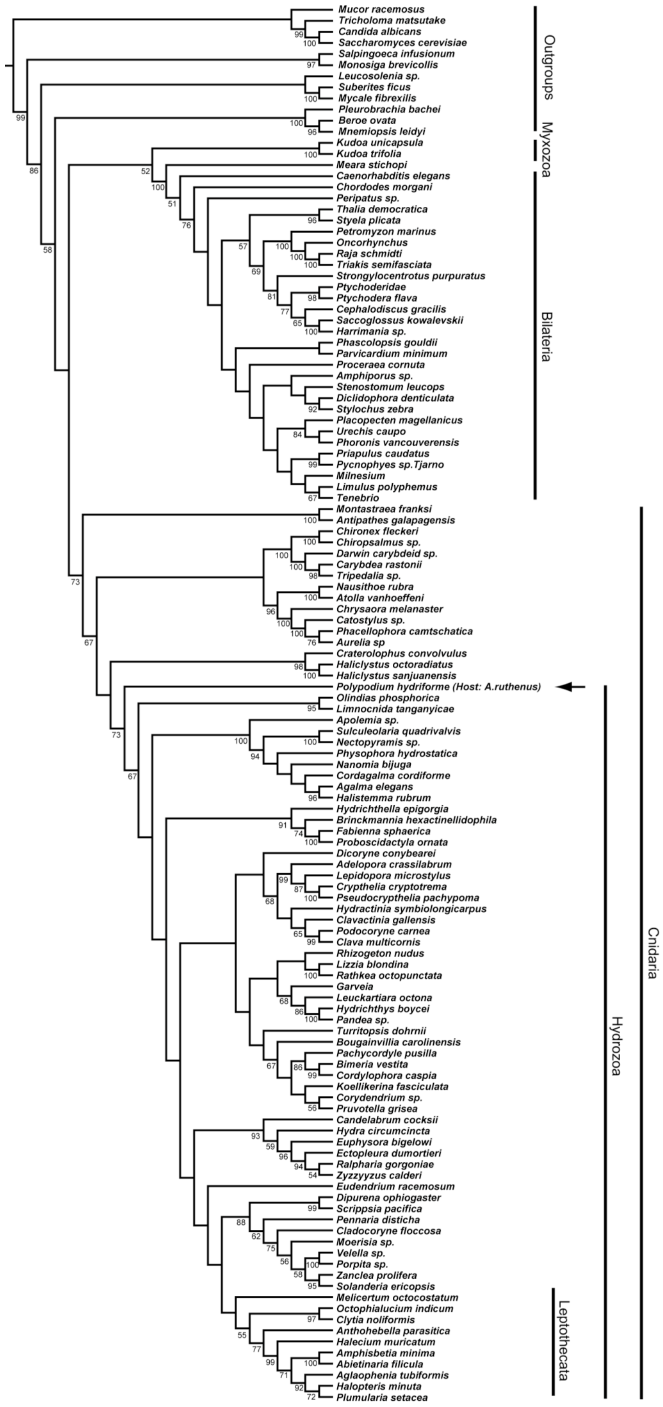

Additional file 1. ML topology of relationships among 126 metazoan taxa, based on a combined analysis of nearly complete 18s and partial 28s rDNA sequences. Arrow indicates *Polypodium* taxa. Bootstrap values greater than 50 are indicated. Corresponding phylogram and assumed model description are displayed in figure 2A.
