# Supplementary material for: Phylogenetic placement of the enigmatic parasite, Polypodium hydriforme, within the Phylum Cnidaria
Source: BMC Evol Biol. 2008 May 9;8:139. doi: 10.1186/1471-2148-8-139 (PMC2396633; doi:10.1186/1471-2148-8-139)
Supplement: Additional file 2 — ML topology of relationships based on partial 28S rDNA sequences. Relationships of 128 metazoan taxa were analyzed with partial 28S rDNA sequences. [file 1471-2148-8-139-S2.pdf]

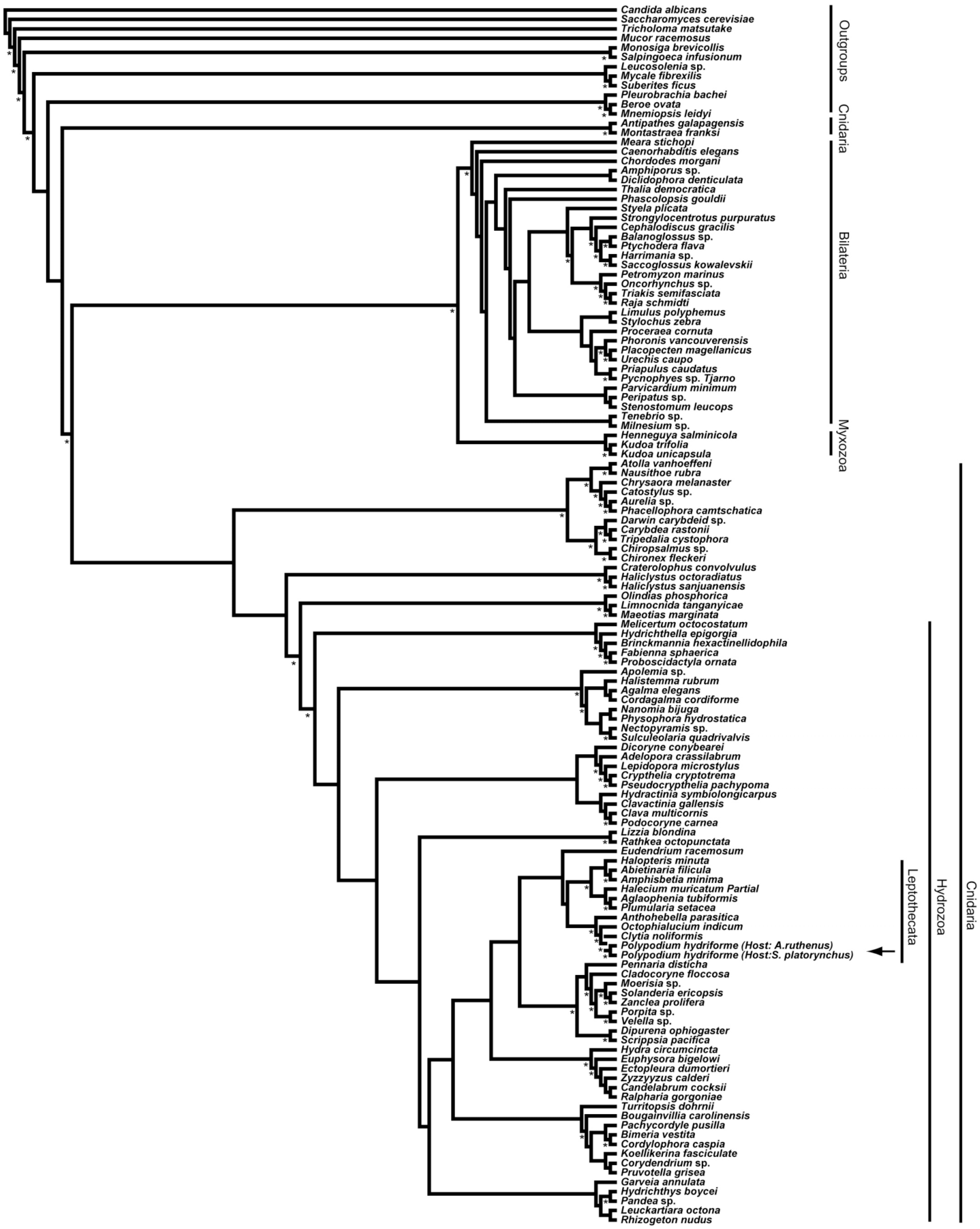

Additional file 2. ML topology of metazoan relationships with partial 28s rDNA sequences with 129 taxa. Bootstrap values greater than 50 are indicated by *, where space permits. Arrow indicates *Polypodium* taxa. The assumed model (GTR+I + G) has six substitutions rates estimated from the data (A-C, 0.8748; A-G, 3.0821; A-T, 1.6210; C-G, 0.6013; C-T, 4.7766; G-T, 1.0000) and an assumed proportion of invariant sites (0.0657) and a gamma shaped parameter or (0.6035).
