# Supplementary material for: Phylogenetic placement of the enigmatic parasite, Polypodium hydriforme, within the Phylum Cnidaria
Source: BMC Evol Biol. 2008 May 9;8:139. doi: 10.1186/1471-2148-8-139 (PMC2396633; doi:10.1186/1471-2148-8-139)
Supplement: Additional file 4 — Parsimony topology of relationships based on 18S rDNA sequences. This parsimony analysis of 18S rDNA sequences included 132 taxa with gaps coded as a fifth state. [file 1471-2148-8-139-S4.pdf]

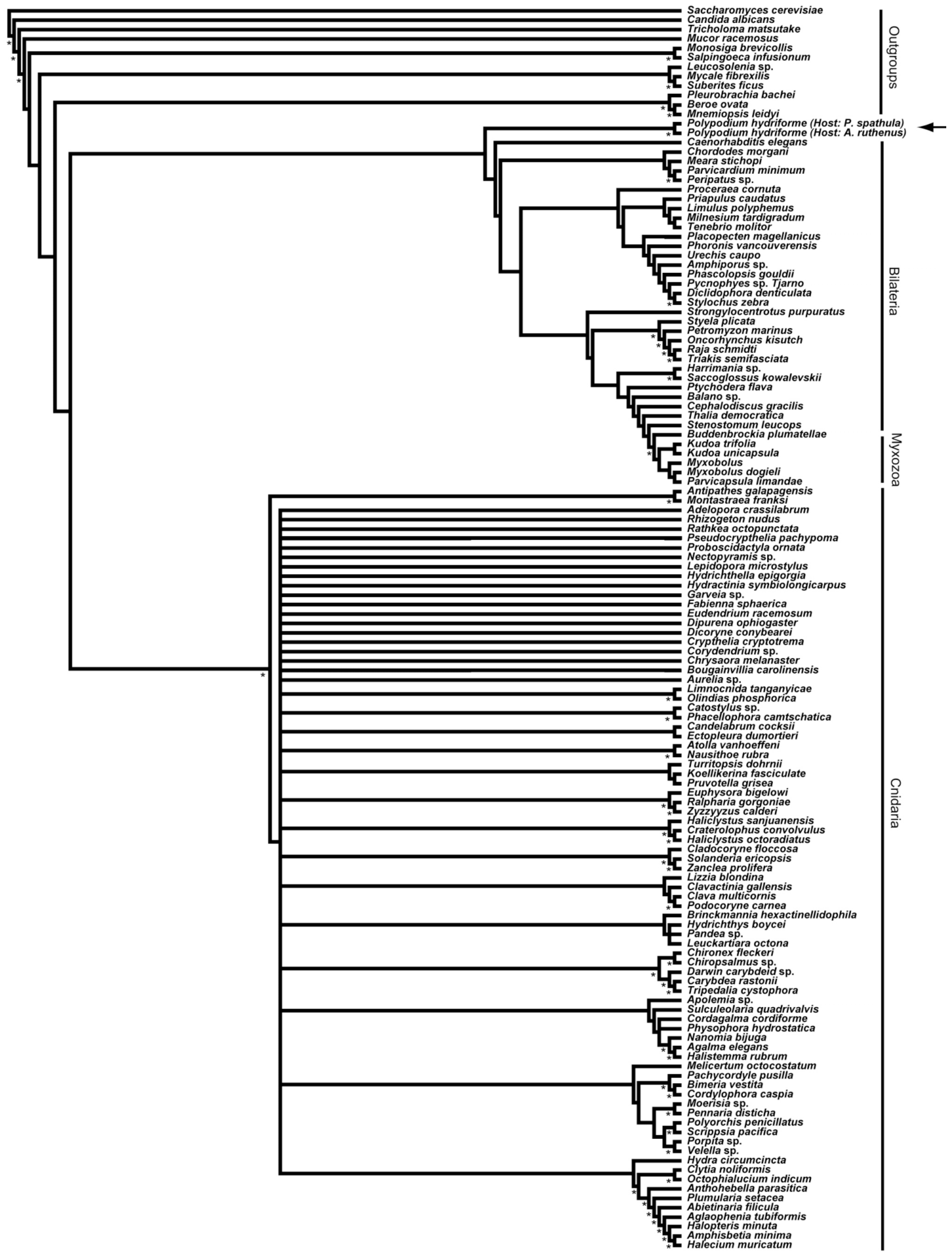


**Additional file 4 -**Parsimony topology of 18s rDNA sequences of 132 taxa with gaps coded as a fifth state. Strict consensus of 120 most parsimonious trees of 17798 steps. Bootstrap values greater than 50 are indicated by *, where space permits.
