# Supplementary material for: Phylogenetic placement of the enigmatic parasite, Polypodium hydriforme, within the Phylum Cnidaria
Source: BMC Evol Biol. 2008 May 9;8:139. doi: 10.1186/1471-2148-8-139 (PMC2396633; doi:10.1186/1471-2148-8-139)
Supplement: Additional file 5 — Parsimony topology of relationships based on 18S rDNA sequences. This parsimony analysis of 18S rDNA sequences included 132 taxa with gaps coded as missing. [file 1471-2148-8-139-S5.pdf]

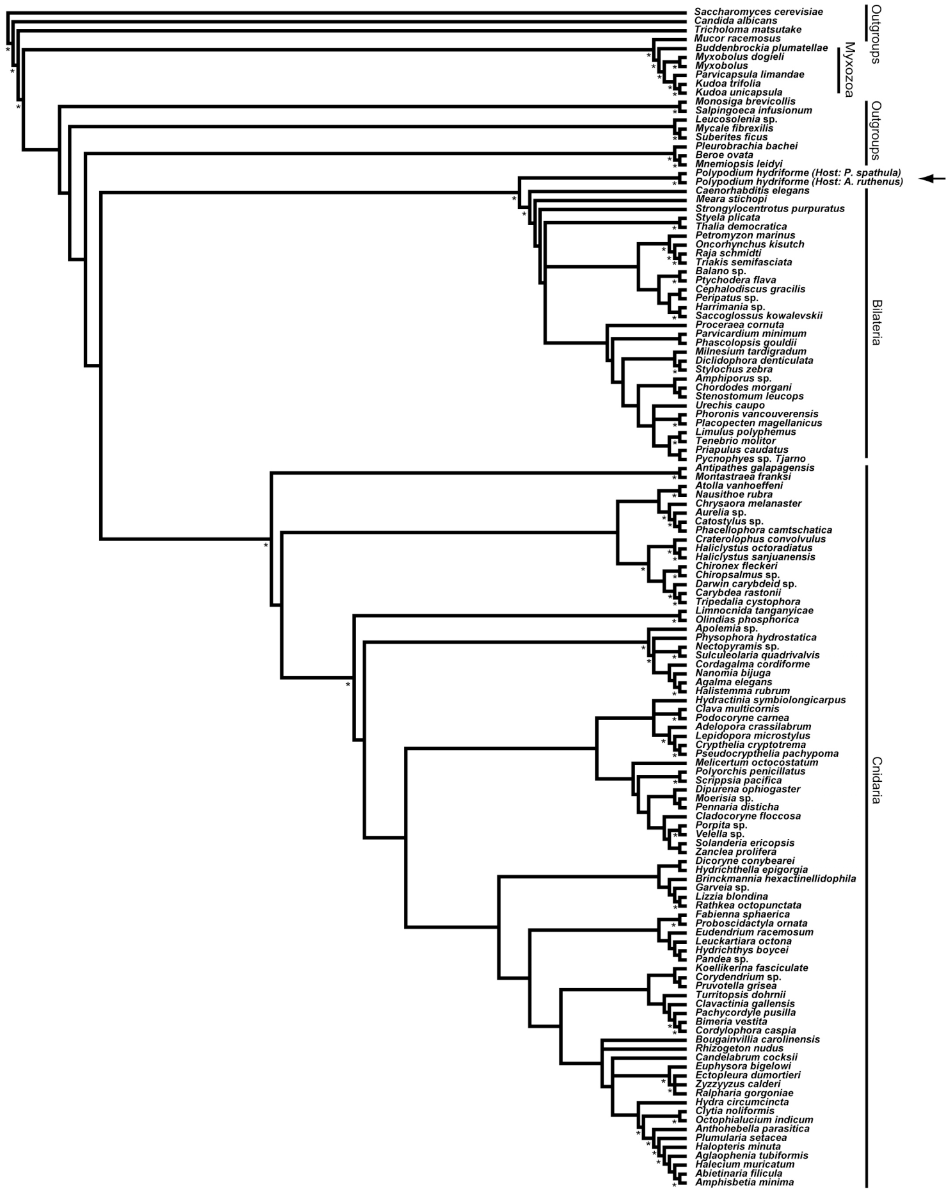

Additional file 5- Parsimony topology of 18s rDNA sequences of 132 taxa with gaps coded as missing. Strict consensus of 40 most parsimonious trees of 11168 steps. Bootstrap values greater than 50 are indicated by *, where space permits.
