# Supplementary material for: Phylogenetic placement of the enigmatic parasite, Polypodium hydriforme, within the Phylum Cnidaria
Source: BMC Evol Biol. 2008 May 9;8:139. doi: 10.1186/1471-2148-8-139 (PMC2396633; doi:10.1186/1471-2148-8-139)
Supplement: Additional file 6 — ML topology of relationships excluding myxozoans, based on partial 28S rDNA data. This ML analysis of partial 28S rDNA sequences excluded myxozoan taxa. [file 1471-2148-8-139-S6.pdf]

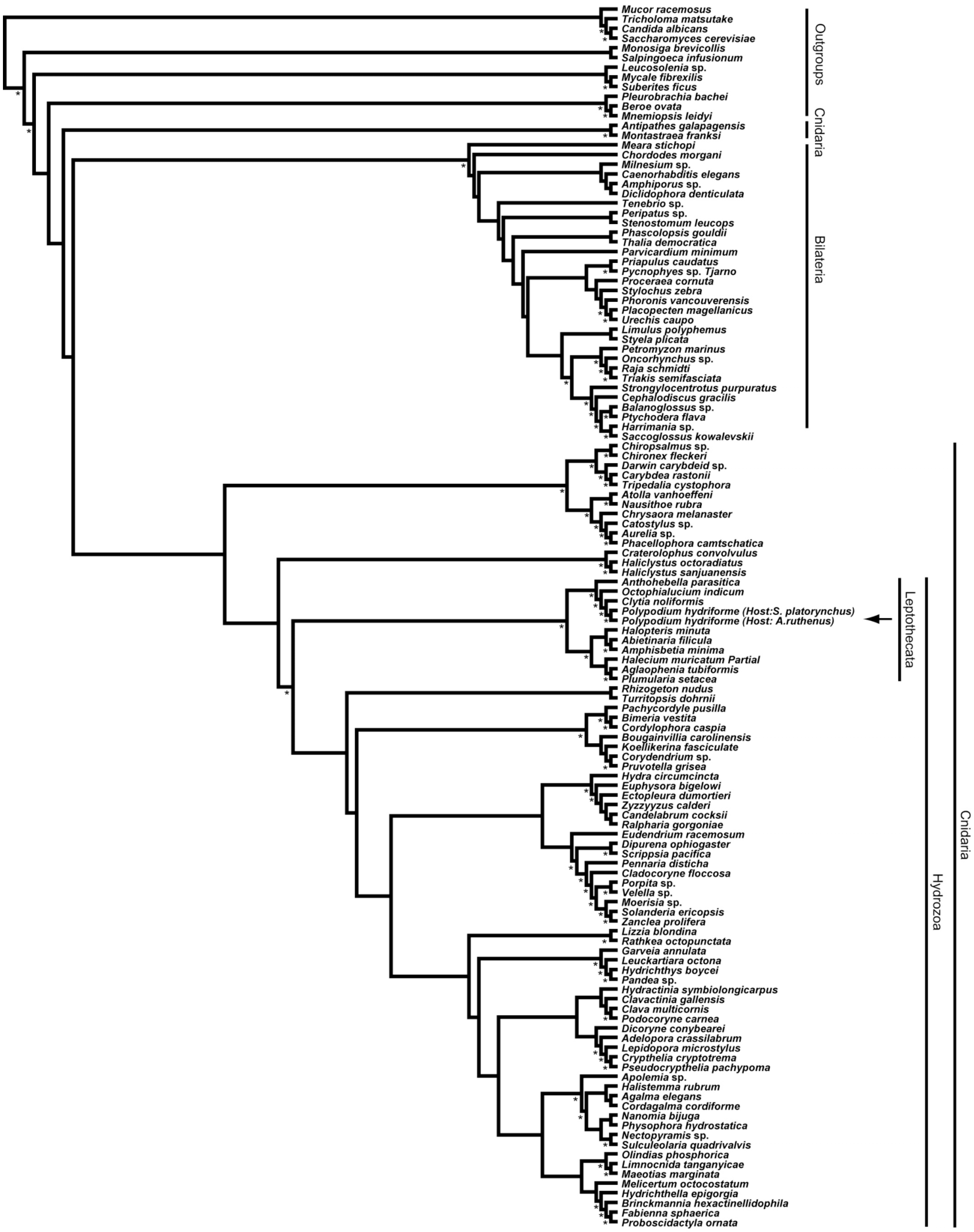

Additional file 6 - ML topology of metazoan relationships with partial 28s rDNA sequences, excluding myzozoan taxa (126 taxa). Bootstrap values greater than 50 are indicated by *, where space permits. Arrow indicates *Polypodium* taxa. The assumed model (GTR+I + G) has six substitutions rates estimated from the data (A-C, 0.9056; A-G, 3.1881; A-T, 1.6535; C-G, 0.5841; C-T, 4.6704; G-T, 1.0000) and an assumed proportion of invariant sites (0.1043) and a gamma shaped parameter or (0.6619).
