# Supplementary material for: Phylogenetic placement of the enigmatic parasite, Polypodium hydriforme, within the Phylum Cnidaria
Source: BMC Evol Biol. 2008 May 9;8:139. doi: 10.1186/1471-2148-8-139 (PMC2396633; doi:10.1186/1471-2148-8-139)
Supplement: Additional file 7 — ML topology of relationships excluding myxozoans, based on combined data. This ML analysis of partial 28S rDNA and 18S sequences excluded myxozoan taxa. [file 1471-2148-8-139-S7.pdf]

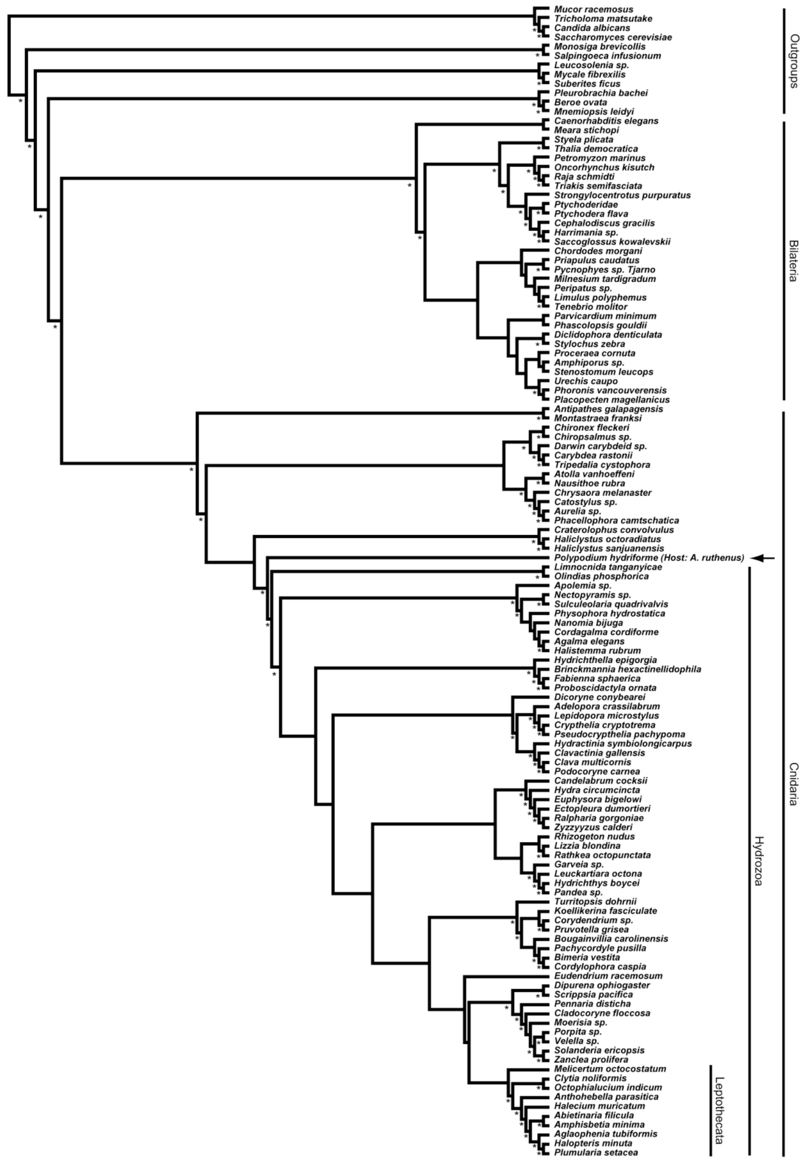

Additional file 7 - ML topology of metazoan relationships of combined partial 28s rDNA and 18s rDNA sequences, excluding myzozoan taxa (124 taxa) . Bootstrap values greater than 50 are indicated by *, where space permits. Arrow indicates *Polypodium* taxa. The assumed model (GTR+I + G) has six substitutions rates estimated from the data (A-C, 1.1915; A-G, 3.4196; A-T, 1.7320; C-G, 0.7425; C-T, 4.758; G-T, 1.0000) and an assumed proportion of invariant sites (0.1860) and a gamma shaped parameter or (0.5577).
