# Supplementary material for: Phylogenetic placement of the enigmatic parasite, Polypodium hydriforme, within the Phylum Cnidaria
Source: BMC Evol Biol. 2008 May 9;8:139. doi: 10.1186/1471-2148-8-139 (PMC2396633; doi:10.1186/1471-2148-8-139)
Supplement: Additional file 8 — ML topology of relationships based on combined data. This analysis of 126 metazoan taxa was based on combined 18S and partial 28S rDNA with length variable sequences removed. [file 1471-2148-8-139-S8.pdf]

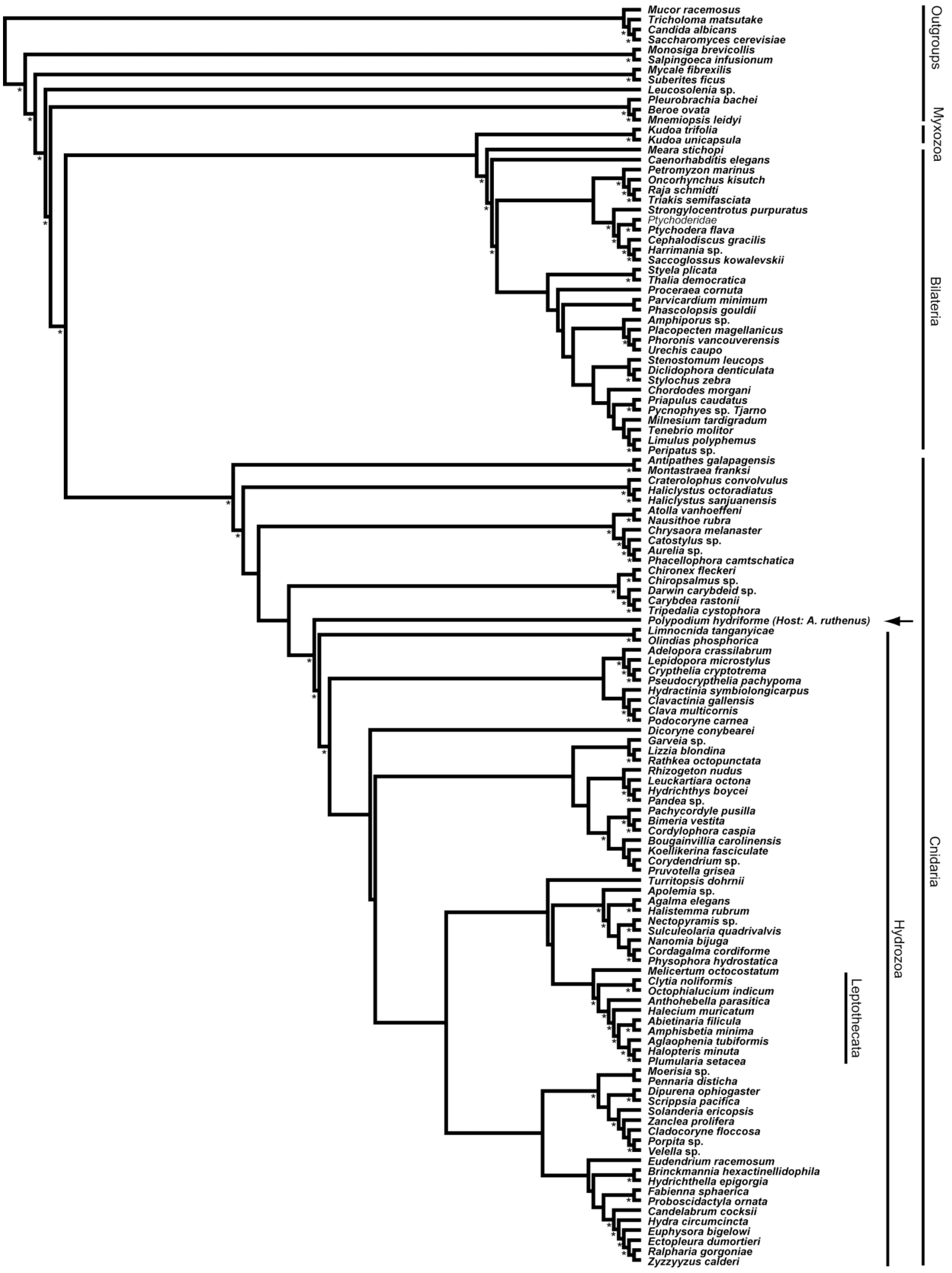


**Additional file 8 -** ML topology of metazoan relationships of combined partial 28s rDNA and 18s rDNA sequences, excluding length-variable regions. The dataset was reduced to 2115 characters (from 4842), 1391 which are parsimony informative (from 2124). Bootstrap values greater than 50 are indicated by *, where space permits. Arrow indicates *Polypodium* taxa. The assumed model (GTR+I + G) has six substitutions rates estimated from the
