# Supplementary material for: Phylogenetic placement of the enigmatic parasite, Polypodium hydriforme, within the Phylum Cnidaria
Source: BMC Evol Biol. 2008 May 9;8:139. doi: 10.1186/1471-2148-8-139 (PMC2396633; doi:10.1186/1471-2148-8-139)
Supplement: Additional file 3 — ML topologies of relationships based on 18S data with and without myxozoans. ML topologies identical to Figure 3 but as cladograms showing bootstrap values. [file 1471-2148-8-139-S3.pdf]

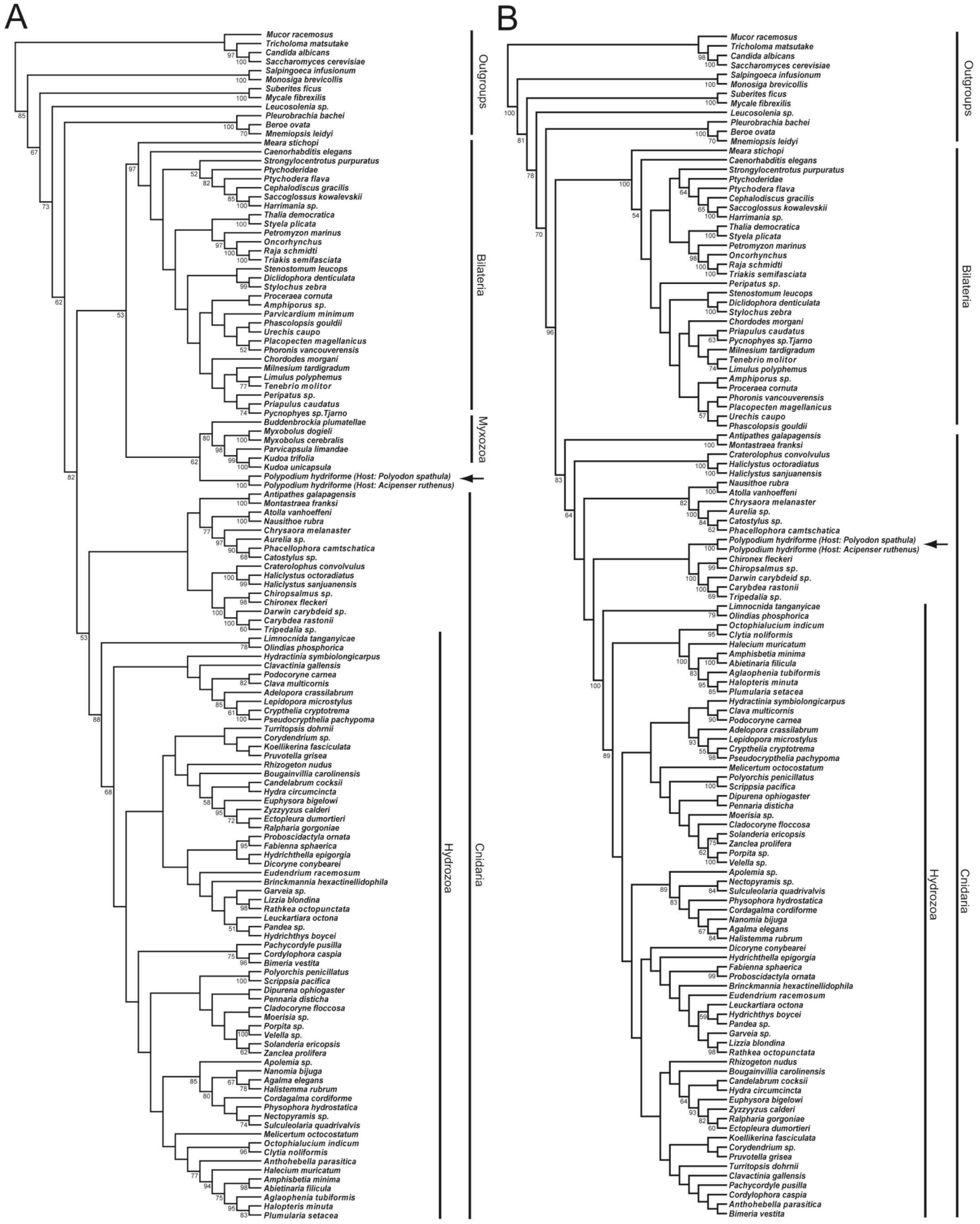

Additional file 3. ML topologies of metazoan relationships of nearly complete 18s rDNA sequences. Arrow indicates *Polypodium* taxa. Bootstrap values greater than 50 are indicated. Corresponding phylograms and assumed model descriptions are displayed in figure 3. A) 132 taxa including 6 myxozoan taxa and two *Polypodium* taxa. B) Same dataset as (A) but with the 6 myxozoan taxa removed.
